# Ticagrelor inverse agonist activity at the P2Y_12_ receptor is non‐reversible versus its endogenous agonist adenosine 5´‐diphosphate

**DOI:** 10.1111/bph.16204

**Published:** 2023-09-01

**Authors:** Jawad Khalil, Tudor Dimofte, Timothy Roberts, Michael Keith, Kumuthu Amaradasa, Matthew S. Hindle, Sukhinder Bancroft, James L. Hutchinson, Khalid Naseem, Thomas Johnson, Stuart J. Mundell

**Affiliations:** ^1^ School of Physiology, Pharmacology and Neuroscience, Faculty of Life Sciences University of Bristol Bristol UK; ^2^ Leeds Institute of Genetics, Health and Therapeutics (LIGHT) University of Leeds Leeds UK; ^3^ Bristol Medical School University of Bristol Bristol UK

**Keywords:** acute coronary syndrome, blood platelets, irreversibility, P2Y_12_ receptor, ticagrelor

## Abstract

**Background and Purpose:**

Ticagrelor is labelled as a reversible, direct‐acting platelet P2Y_12_ receptor (P2Y_12_R) antagonist that is indicated clinically for the prevention of thrombotic events in patients with acute coronary syndrome (ACS). As with many antiplatelet drugs, ticagrelor therapy increases bleeding risk in patients, which may require platelet transfusion in emergency situations. The aim of this study was to further examine the reversibility of ticagrelor at the P2Y_12_R.

**Experimental Approach:**

Studies were performed in human platelets, with P2Y_12_R‐stimulated GTPase activity and platelet aggregation assessed. Cell‐based bioluminescence resonance energy transfer (BRET) assays were undertaken to assess G protein‐subunit activation downstream of P2Y_12_R activation.

**Key Results:**

Initial studies revealed that a range of P2Y_12_R ligands, including ticagrelor, displayed inverse agonist activity at P2Y_12_R. Only ticagrelor was resistant to washout and, in human platelet and cell‐based assays, washing failed to reverse ticagrelor‐dependent inhibition of ADP‐stimulated P2Y_12_R function. The P2Y_12_R agonist 2MeSADP, which was also resistant to washout, was able to effectively compete with ticagrelor. In silico docking revealed that ticagrelor and 2MeSADP penetrated more deeply into the orthosteric binding pocket of the P2Y_12_R than other P2Y_12_R ligands.

**Conclusion and Implications:**

Ticagrelor binding to P2Y_12_R is prolonged and more akin to that of an irreversible antagonist, especially versus the endogenous P2Y_12_R agonist ADP. This study highlights the potential clinical need for novel ticagrelor reversal strategies in patients with spontaneous major bleeding, and for bleeding associated with urgent invasive procedures.

**Abbreviations**
ACD: acid citrate dextrose
ACS: acute coronary syndrome
ANOV: analysis of variance
BRET: bioluminescence resonance energy transfer
BSA: bovine serum albumin
BUDE: Bristol University Docking Engine
DiOC_6_(3): 3,3′‐dihexyloxacarbocyanine iodide
DMEM: Dulbecco's modified Eagle's medium
DTT: dithiothreitol
EDTA: ethylenediaminetetraacetic acid
FBS: fetal bovine serum
HEK 293: human embryonic kidney 293
HEPES: *N*‐2‐hydroxyethylpiperazine‐*N*9‐2‐ethanesulfonic acid
MD: molecular dynamic
P2Y_12_R: P2Y_12_ receptor
PLATO: Platelet Inhibition and Patient Outcomes
PPP: platelet‐poor plasma
PRP: platelet‐rich plasma
RBC: red blood cell
RMSD: root‐mean‐square deviation
SEM: standard error of the mean

**What is already known**

- Ticagrelor has been labelled as a reversible, direct‐acting, platelet P2Y_12_R antagonist.
- Ticagrelor is indicated clinically for prevention of thrombotic events in patients with acute coronary syndrome.

**What does this study add**

- Ticagrelor is an inverse agonist at the P2Y_12_R and resistant to washout in human platelets.
- Ticagrelor penetrates more deeply into the orthosteric binding pocket of P2Y_12_R than other P2Y_12_R ligands.

**What is the clinical significance**

- Current clinical guidelines in patients on P2Y_12_R antagonists should be reconsidered, especially for ticagrelor.
- Where clinically feasible, ticagrelor administration should cease at least 5 days before major non‐cardiac surgery.

## 1 INTRODUCTION

Since its introduction, in 2011, the antiplatelet agent ticagrelor has established itself as a standard of care in the management of patients with acute coronary syndrome (ACS) (Collet et al., 2021). The drug selectively binds to the P2Y_12_ receptor (P2Y_12_R) at the platelet surface and provides faster, greater, and more consistent platelet inhibition when compared with other antiplatelet drugs, including clopidogrel (Van Giezen et al., 2009; Wallentin et al., 2009). Unlike the thienopyridine‐based and orally administered P2Y_12_R antagonists (clopidogrel, ticlopidine and prasugrel), all of which are prodrugs requiring hepatic metabolism to produce active compounds that bind irreversibly to P2Y_12_R to exert anti‐aggregatory activity, ticagrelor is a non‐thienopyridine (cyclopentyl‐triazolopyrimidine) not requiring bioactivation to act on P2Y_12_R (Butler & Teng, 2010). In addition, ticagrelor is an inverse agonist at the endogenous P2Y_12_R on blood platelets (Aungraheeta et al., 2016; Garcia et al., 2019). Ticagrelor, as an inverse agonist, can reduce agonist‐independent receptor activity (De Ligt et al., 2000). In the Platelet Inhibition and Patient Outcomes (PLATO) trial, ticagrelor's more potent platelet inhibition provided greater clinical benefit with a decreased risk of major adverse cardiovascular events and improved survival in patients with ACS, when compared with clopidogrel (Wallentin et al., 2009). However, similar to prasugrel, ticagrelor is associated with an increased risk of major bleeding, which may persist for days after drug discontinuation (Wallentin et al., 2009; Wiviott et al., 2007). The management of bleeding risk represents a major clinical challenge, especially in patients who present with spontaneous life‐threatening bleeding or who require urgent surgical procedures, because there are no standardized reversal strategies or clinically available antidotes (Buchanan et al., 2015).

In the absence of specific antidotes, platelet transfusion often is used in an emergency to reverse the effect of antiplatelet drugs (Sousa‐Uva et al., 2014). In the absence of platelet transfusion, the offset of ticagrelor activity effects is approximately 5 days (Gurbel et al., 2009). This approach is primarily based on the hypothesis that substituting drug‐inhibited platelet populations with functional donor platelets could result in overall improved haemostatic response. Notably, platelet transfusion efficiently reverses the inhibitory effect of clopidogrel and prasugrel in a dose‐dependent manner (Bonhomme et al., 2015; Li et al., 2012; Schoener et al., 2017). However, the effectiveness of this approach with ticagrelor has been questioned in a number of recent studies (Godier et al., 2015; Trenk et al., 2019; Willeman et al., 2018; Zhang et al., 2019). Persistent inhibition of platelet aggregation is observed for several days after discontinuation of ticagrelor, and can still be observed when plasma concentrations of ticagrelor are undetectable, with platelet reactivity only returning to near‐normal levels about 5 days following cessation of treatment (Storey et al., 2011).

In this study, we sought to further probe the reversibility of ticagrelor binding to P2Y_12_R and compared it against other receptor antagonists. Importantly, we found that the endogenous P2Y_12_R agonist ADP was unable to restore P2Y_12_R activity following ticagrelor treatment, even after extensive washing of antagonist, either when using cell lines or human platelets.

## 2 METHODS

### 2.1 Materials

FLAG‐tagged human wild‐type P2Y_12_R constructs using pcDNA3.1 were generated as previously described (Hardy et al., 2005), and their validity was confirmed with sequencing (Eurofins Genomics). The P2Y_12_R ligands AR‐C66096 tetrasodium salt, cangrelor (AR‐C66931MX), ticagrelor, elinogrel, AZD1283 and the adenylyl cyclase activator forskolin were procured from Tocris Bioscience (Bristol, UK). Prasugrel (R‐138727) was obtained from Eli Lilly Research Laboratories (Indianapolis, IN). Luciferase substrate (Coelenterazine 400a) was obtained from Insight Biotechnology Limited (Wembley, UK). Cell culture reagents including Dulbecco's modified Eagle's medium (DMEM), fetal bovine serum (FBS), penicillin–streptomycin and Lipofectamine 2000 were from Invitrogen (Paisley, UK). All other reagents were purchased from Sigma‐Aldrich.

### 2.2 Ethics

Approval of this study was granted by the South Central—Hampshire A Research Ethics Committee (NHS‐REC Reference 20/SC/0222).

### 2.3 Isolation of human platelets

Samples were obtained from healthy consenting male and female volunteers, who confirmed that they had not received any medication that affects platelet activity. Whole blood was collected in 4% sodium citrate and acid citrate dextrose (ACD) (29.9‐mM Na_3_C_6_H_5_O_7_, 113.8‐mM glucose, 72.6‐mM NaCl and 2.9‐mM citric acid [pH 6.4]). Platelet‐rich plasma (PRP) was obtained by centrifugation at 180 *g* for 17 min, treated with 0.02‐U·ml^−1^ apyrase and 10‐μM indomethacin and then centrifuged at 550 *g* for 10 min. The platelet pellet was then resuspended in wash buffer (36‐mM citric acid, 10‐mM ethylenediaminetetraacetic acid [EDTA], 5‐mM glucose, 5‐mM KCl and 9‐mM NaCl) containing 0.02‐U·ml^−1^ apyrase and 10‐μM indomethacin and centrifuged at 550 *g* for 10 min. Platelets (1 × 10^9^/ml) were resuspended in modified Tyrode's buffer (150‐mM NaCl, 5‐mM *N*‐2‐hydroxyethylpiperazine‐*N*9‐2‐ethanesulfonic acid [HEPES], 0.55‐mM NaH_2_PO_4_, 7‐mM NaHCO_3_, 2.7‐mM KCl, 0.5‐mM MgCl_2_ and 5.6‐mM glucose [pH 7.4]) supplemented with 0.02‐U·ml^−1^ apyrase and 10‐μM indomethacin and rested at 30°C for at least 30 min before experimentation. All platelet preparations were processed at room temperature (19–23°C).

### 2.4 Platelet aggregation

PRP was pre‐treated either with AR‐C66096 (10 μM), ticagrelor (10 μM) or vehicle (0.1% dimethyl sulfoxide [DMSO]). In studies where drug removal was attempted by washing, platelets were washed by centrifugation and the platelet pellet was resuspended in fresh 300‐μl platelet‐poor plasma (PPP). Platelets were incubated for 10 min between wash steps, except in those experiments assessing long time periods of washout where platelets were incubated after the second wash step for 1, 4 or 24 h at 37°C and kept in suspension by gentle agitation on a rocker. Washed platelet samples were directly compared with time‐matched drug/DMSO‐treated controls (i.e., 24‐h washout compared with 24‐h ticagrelor treatment). In all cases, platelet aggregation was initiated by 10‐μM ADP under constant stirring conditions (1000 r.p.m.) for 5 min at 37°C and assessed using a light transmission aggregometry with a CHRONO‐LOG 700 aggregometer (Labmedics, Manchester, UK).

### 2.5 Platelet viability assay

Platelet samples were stored for the indicated time points at a platelet count of 3 × 10^8^/ml at 37°C with orbital shaking (20 r.p.m.). Annexin V exposure was measured as described previously (Hindle et al., 2021). Briefly, at each time point, a sample was incubated with annexin V‐APC (Thermo Fisher R37176) and CD42b‐FITC (BD Biosciences 555472; RRID:AB_395864) for 20 min in modified Tyrode's buffer supplemented with 2‐mM CaCl_2_ to facilitate annexin V binding; EDTA (10 mM) was included in control samples to establish background binding. At 20 min, samples were fixed in 1% paraformaldehyde (v/v) and CD42b‐positive platelet events were collected for 2.5 min at a 10‐μl·min^−1^ flow rate to measure annexin V expression and allow calculation of events/μl. Samples were assessed on a Beckman Coulter CytoFLEX Flow Cytometer using two lasers (488 and 638 nm) and two detectors (525/40 BP and 660/10 BP). Viability was calculated as inverse annexin V exposure.

### 2.6 In vitro platelet flow assays

Whole blood was collected in citrate vacutainers and treated either with P2Y_12_R inhibitors or vehicle as indicated for 10 min before isolation of PRP, alongside further untreated tubes from which both PRP and red blood cell (RBC) fractions were retained. Platelets were isolated by centrifugation in the presence of ACD, whilst PPP was prepared from the untreated samples in the absence of ACD using two successive 10‐min spins of 500 *g* to minimize platelet contamination. Platelets were washed in CGS buffer before resuspension in PPP to 5 × 10^8^/ml and resting for 30 min. RBC fraction was resuspended to full volume with PPP and spun as per PRP fractionation to reduce residual platelet numbers. Whole blood was reconstituted from the reconstituted PRP and RBC fractions at a volume ratio of 2:3, to give a final platelet concentration of 2 × 10^8^/ml, and loaded with 2‐μM 3,3′‐dihexyloxacarbocyanine iodide (DiOC_6_(3)). Blood samples were incubated either with fresh inhibitors or vehicle as indicated for 10 min at 37°C before flowing through collagen‐coated, bovine serum albumin (BSA)‐blocked μ‐slide VI 0.1 channels in parallel for 10 min at 37°C, at 6 ml·h^−1^, giving a calculated shear rate of 1000/s. This was followed by 10‐min flow with 4% paraformaldehyde. Samples were perfused with ibidi mounting medium and imaged using a 20× dry objective on a Leica SP5II confocal scanning microscope, illuminated at 488 nm. Three stacked images at 1‐μm z spacing were taken from the beginning, middle and end of each channel, respectively. Surface area coverage and thrombus volume were calculated using Fiji/ImageJ and channel data were averaged.

### 2.7 GTPase activity assay

Washed platelet suspension (1 × 10^9^/ml) was either treated or untreated with different agonist/antagonists at indicated time points, and reactions were stopped with an equal volume of fractionation buffer (320‐mM sucrose, 4‐mM HEPES and 0.5‐mM Na_3_PO_4_, pH 7.4) supplemented with a protease inhibitor cocktail. Samples were subjected to five freeze–thaw cycles in liquid nitrogen. Unbroken platelets were removed by centrifugation at 5000 *g* for 5 min at 4°C before ultracentrifugation at 180,000 *g* for 90 min at 4°C. The supernatant was removed, and the pellet fraction was washed two times with 1 ml of fractionation buffer and resuspended in 50 μl of GTPase assay buffer. The intrinsic GTPase activity of Gαi in human platelets was measured using the GTPase‐Glo assay (Promega). Briefly, the membrane fraction of human washed platelets was resuspended in assay buffer consisting of 20‐mM HEPES (pH 7.4), 100‐mM NaCl, 10‐mM MgCl_2_ and 1‐mg·ml^−1^ BSA and treated either in the presence or absence of different agonists/antagonists at indicated time points before incubation with GTP (2 μM) and dithiothreitol (DTT) (1 mM) for 1 h at 24°C. Then GTPase‐Glo reagent, including 5‐μM ADP, was added, briefly mixed and incubated for 30 min with shaking at 24°C. Finally, detection reagent was added for 5 min in the dark and GTP hydrolysis (luminescence) was measured using a Tecan Infinite M200 Pro microplate reader (Männedorf, Switzerland).

### 2.8 Cell culture and transfection

Human embryonic kidney (HEK 293T; RRID:CVCL_0063) cells were maintained in DMEM and supplemented with 10% FBS, 100‐U·ml^−1^ penicillin and 100‐μg·ml^−1^ streptomycin at 37°C in a humidified atmosphere of 95% air and 5% CO_2_. Cells were grown in 100‐mm dishes to 70%–90% confluence and transiently co‐transfected with 1‐ to 1.5‐μg DNA using Lipofectamine 2000 according to manufacturer's protocol. Briefly, cells were incubated with DNA/Lipofectamine complexes for 5 h, the media were replaced and cells were analysed for bioluminescence resonance energy transfer 2 (BRET2) assay after 48‐h transfection.

### 2.9 Bioluminescence resonance energy transfer (BRET) measurement

To investigate the effect of a drug on heterotrimeric G‐protein activation, vectors encoding N‐terminal FLAG‐P2Y_12_R, RlucII‐Gαi1 (a mutated brighter version of the Rluc), Gβ1 and GFP2‐Gγ2 were transiently co‐transfected into HEK 293T cells as previously described (Zhao et al., 2021). In brief, 48 h after transfection, cells were detached using phenol‐free Trypsin–EDTA, washed 2× and resuspended in phenol‐free DMEM at room temperature. Then 80 μl of 100,000 cells per well was transferred to a 96‐well white flat‐bottom microplate (Greiner Bio‐One, Austria) and cells were rested for at least 30 min at 37°C before experimentation. Cells were then treated with or without different concentrations of ligands at indicated time points. BRET2 signal between RlucII and green fluorescent protein (GFP) was measured immediately, following the addition of Coelenterazine 400a (luciferase substrate) at a final concentration of 2 μM using a FLUOstar® Omega plate reader (BMG Labtech, UK). The BRET signal was calculated as the ratio of the light emitted by acceptor (GFP2) (510–540 nm) to donor (RlucII) (410–480 nm). To determine the delta BRET (ΔBRET), the value obtained in the vehicle condition was subtracted from the one measured with ligand.

### 2.10 In silico ligand docking and molecular dynamic (MD) simulations

ADP, 2MeSADP, ticagrelor and cangrelor were docked as outlined below. ADP and 2MeSADP were docked within the agonist‐bound crystal model of the receptor (Protein Data Bank [PDB] ID: 4PXZ; Zhang, Zhang, Gao, Paoletta, et al., 2014). The antagonists, ticagrelor and cangrelor, were docked within the antagonist‐bound crystal structure of P2Y_12_R (PDB ID: 4NTJ; Zhang, Zhang, Gao, Zhang, et al., 2014). Before docking, 10,000 conformations of each ligand were generated to take account for ligand flexibility.. To generate these ligand conformations, MD simulations of each ligand were produced. Solvated in a box of TIP3P H_2_O and 0.15‐M NaCl, each ‘free‐in‐solution’ ligand was simulated for 1 μs, employing AMBER GAFF force fields. The 10,000 ligand conformations generated were docked using the Bristol University Docking Engine (BUDE) within the appropriate crystal model of P2Y_12_R, and the free energy of binding predicted. Based on the free energy and 125‐ns MD simulations of several ligand–receptor complexes, a final ligand–receptor complex was selected for each ligand. For MD simulation of the ligand–receptor complex, the complex was embedded in a membrane of POPC and 20% cholesterol and solvated in TIP3P H_2_O with 0.15‐M NaCl. The system was minimized over 10,000 steps and then heated in two steps, first to 100,000 and then to 310,000. The heated system was then equilibrated over 10 rounds of 500‐ps simulations, under anisotropic pressure scaling. Subsequent simulation was conducted in the presence of ff14SB, GAFF and Lipid14 force fields, using the Langevin thermostat and anisotropic Berendenson barostat. With a timestep of 0.002 ps, coordinates were written to the trajectory file every 100 ps. Based on root‐mean‐square deviation (RMSD) analysis of the stability of the ligand–receptor complex during the 125‐ns MD simulation, a final binding pose for each ligand was selected. The plane of the extracellular membrane of the ligand–receptor simulation system was used to calculate the depth of each ligand within P2Y_12_R. The distance of the deepest point of each ligand and the geometric centre of each ligand, from the extracellular membrane plane, was calculated. The initial docked poses were used for the distance calculations.

### 2.11 Data and statistical analysis

Results are presented as average ± standard error of the mean (SEM) of at least five independent experiments. Data analyses were performed using GraphPad Prism 8 (San Diego, CA). One‐way analysis of variance (ANOVA), unless otherwise specified, was used to detect statistically significant differences with Bonferroni post hoc analysis applied for multiple comparisons. A *P* value less than 0.05 was considered significant. The data and statistical analysis comply with the recommendations of the *British Journal of Pharmacology* on experimental design and analysis in pharmacology (Curtis et al., 2022).

### 2.12 Nomenclature of targets and ligands

Key protein targets and ligands in this article are hyperlinked to corresponding entries in https://www.guidetopharmacology.org and are permanently archived in the Concise Guide to PHARMACOLOGY 2021/22 (Alexander et al., 2021).

## 3 RESULTS

**FIGURE 1:**
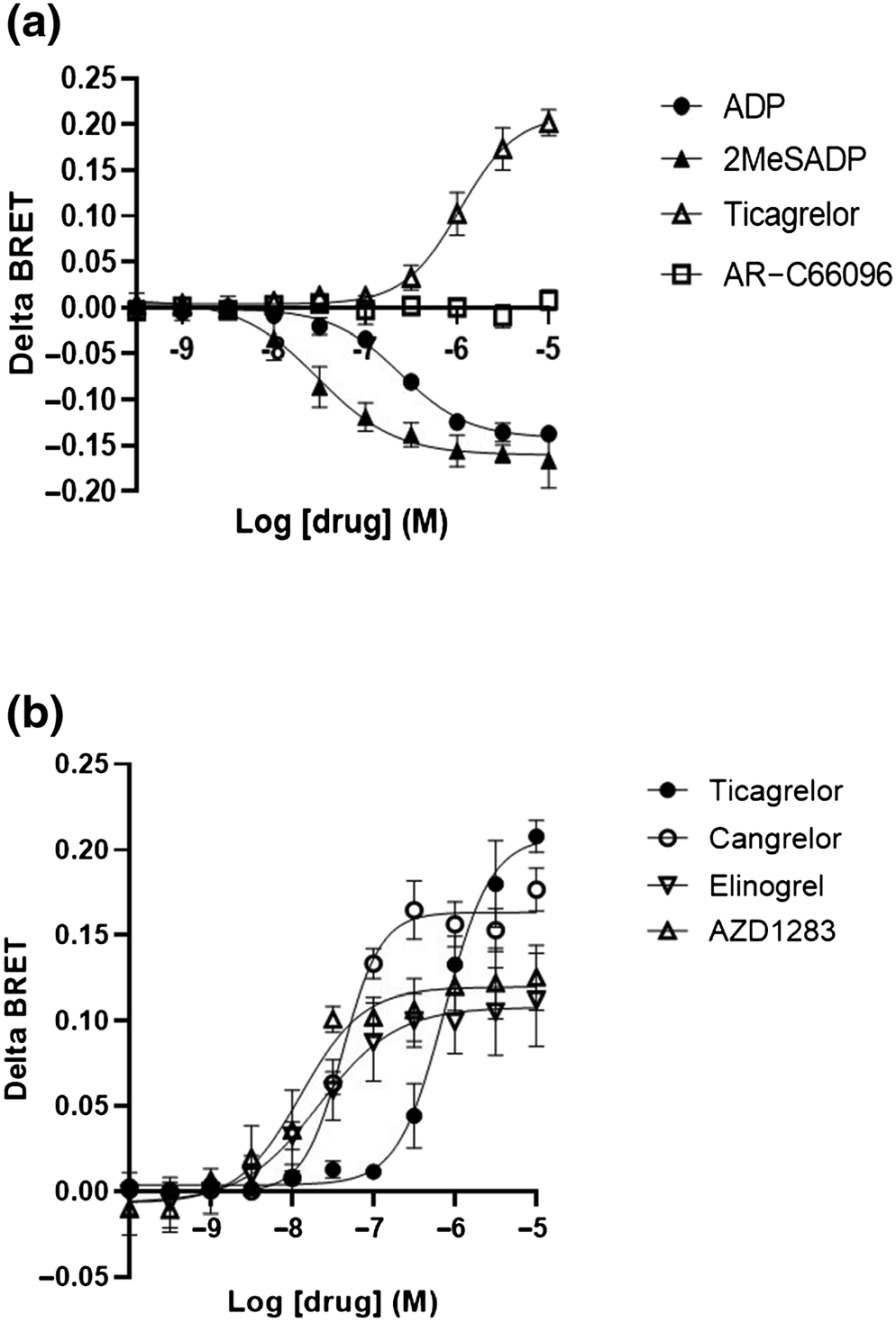
Ligand‐dependent regulation of P2Y_12_R responsiveness as assessed by a BRET‐based assay. HEK 293T cells were co‐transfected with human FLAG‐P2Y_12_R and heterotrimeric G proteins, RlucII‐Gαi, untagged Gβ and GFP‐Gγ. Forty‐eight hours after transfection, cells were treated with ligand for 5 min at 37°C and subsequent changes in BRET signal were measured with reduction in BRET signal, a consequence of G protein‐subunit disassociation. Results are expressed as delta BRET. (a) Ligand‐induced changes in receptor activation as assessed by BRET following treatment with ticagrelor, 2MeSADP, ADP and AR‐C66096 versus vehicle (0.1% DMSO) control. (b) Ligand‐induced changes in receptor activation as assessed by BRET following treatment with ticagrelor, cangrelor, elinogrel and AZD1283 versus vehicle (0.1% DMSO) control. Data shown are the means ± SEM of at least five independent experiments, each performed in triplicate.

Our initial experiments aimed to recapitulate our previous findings demonstrating that ticagrelor has inverse agonist activity at the P2Y_12_R (Aungraheeta et al., 2016). In these studies, we used a standard BRET‐based approach that measures agonist‐stimulated changes in Gα/βγ disassociation using the functionally validated BRET pair RlucII‐Gαi1 and GFP10‐Gγ2 (Gales et al., 2005; Zhao et al., 2021). As expected, receptor activation with P2Y_12_R agonists ADP or 2MeSADP promoted rapid RlucII‐Gαi1 and GFP10‐Gγ2 disassociation leading to a decrease in ΔBRET signal in a concentration‐dependent manner (Figure 1a). In agreement with our previous studies (Aungraheeta et al., 2016) and those of Garcia et al. (2019), ticagrelor decreased RlucII‐Gαi1 and GFP10‐Gγ2 disassociation leading to an increase in ΔBRET signal, whereas the neutral P2Y_12_R antagonist AR‐C66096 had no effect. We subsequently assessed if a range of P2Y_12_R antagonists, including clinically used drugs such as cangrelor or experimental compounds including elinogrel and AZD1283, had inverse agonist activity at the P2Y_12_R (Figure 1b). Interestingly, all these compounds did indeed display a degree of inverse agonism although with a range of potencies and *E*_max_ values (see Table 1).

**TABLE 1:** Potency (nM) and *E*_max_ inverse agonist activity values of a range of P2Y_12_R ligands as assessed by BRET‐based assay data taken from Figure 1b.

Drug	EC_50_ (nM)	*E*_max_ (AU)
Ticagrelor	744.3 (551.0–1180.0)	0.2076 (0.1864–0.2410)
Cangrelor	41.5 (32.8–53.1)	0.1634 (0.1541–0.1730)
Elinogrel	21.4 (12.1–38.6)	0.1081 (0.09770–0.1220)
AZD1283	12.7 (8.3–19.1)	0.1197 (0.1110–0.1302)

*Note*: Data represent the means of at least five independent experiments, each performed in triplicate with numbers in brackets representing 95% confidence intervals.

Abbreviations: BRET, bioluminescence resonance energy transfer; P2Y_12_R, P2Y_12_ receptor.

**FIGURE 2:**
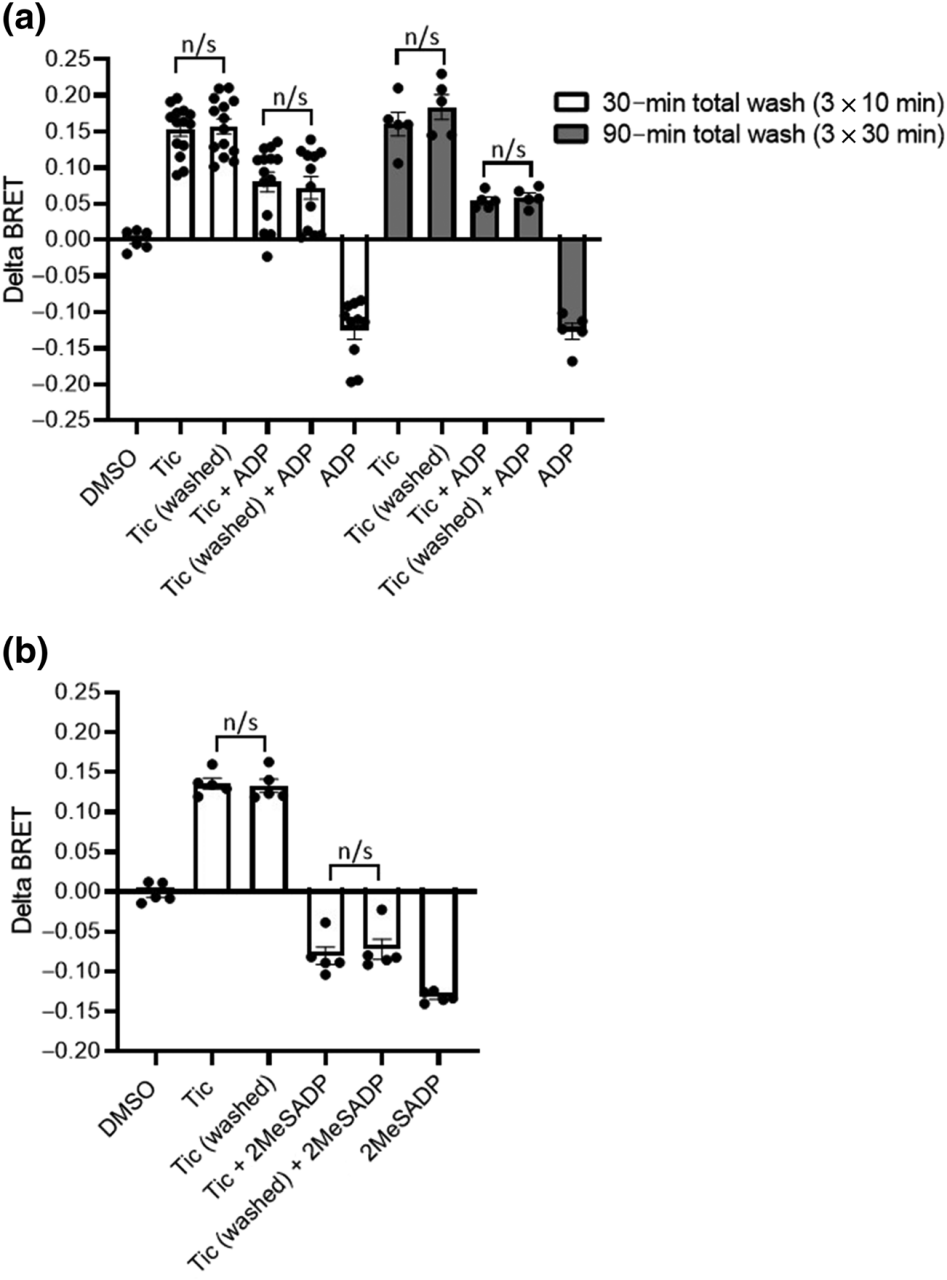
Ticagrelor‐dependent activity at the P2Y_12_R is resistant to washout. HEK 293T cells were co‐transfected with human FLAG‐P2Y_12_R and heterotrimeric G proteins, RlucII‐Gαi, untagged Gβ and GFP‐Gγ. Forty‐eight hours after transfection, cells were treated with ticagrelor (10 μM) or vehicle control for 30 min at 37°C. Following ticagrelor treatment, cells were washed (Tic washed) for either 3 × 10‐min washes (a, b) or 3 × 30‐min washes (a), and receptor activity was compared with that following acute ticagrelor treatment (10 μM; 5 min; Tic). In (a), ADP‐stimulated (10 μM; 5 min) activity was assessed in non‐ticagrelor‐treated cells (ADP), in cells treated acutely with ticagrelor (Tic + ADP) or in cells following more prolonged ticagrelor treatment and subsequent washing (Tic washed + ADP). In (b), 2MeSADP‐stimulated (10 μM; 5 min) activity was assessed in non‐ticagrelor‐treated cells (2MeSADP), in cells treated acutely with ticagrelor (Tic + 2MeSADP) or in cells following more prolonged ticagrelor treatment and subsequent washing (Tic washed + 2MeSADP). Data shown are the means ± SEM of at least five independent experiments, each performed in triplicate. Statistical analysis was performed using one‐way ANOVA and followed by Bonferroni's multiple comparison test.

As outlined above, there is still considerable controversy regarding the reversibility of ticagrelor at the P2Y_12_R versus ADP, the endogenous agonist at this receptor. We therefore sought to examine this using our BRET‐based approach (Figure 2a). Transfected cells were treated with ticagrelor (10 μM; 10 min) or vehicle control. Ticagrelor reversibility was assessed by washing cells with either 3 × 10‐min washes (30 min total) or 3 × 30‐min washes (90 min total). Ticagrelor activity, either inverse agonism in the absence of ADP or antagonism of ADP (10 μM)‐stimulated P2Y_12_R activity, was subsequently assessed. In the absence of washes, ticagrelor inverse agonism was evident whilst ADP‐stimulated P2Y_12_R activity was completely attenuated by ticagrelor pre‐treatment. Notably, neither the shorter nor more extended wash protocols were able to effectively reverse either ticagrelor inverse agonism or antagonism of ADP‐stimulated P2Y_12_R activity. Further study was undertaken comparing ticagrelor with the potent P2Y_12_R agonist 2MeSADP (10 μM; Figure 2b). Notably, 2MeSADP was more effective than ADP in reversing ticagrelor inverse agonism in unwashed cells. However, as in Figure 2a, ticagrelor antagonism of 2MeSADP‐stimulated P2Y_12_R activity was unchanged by washing cells.

**FIGURE 3:**
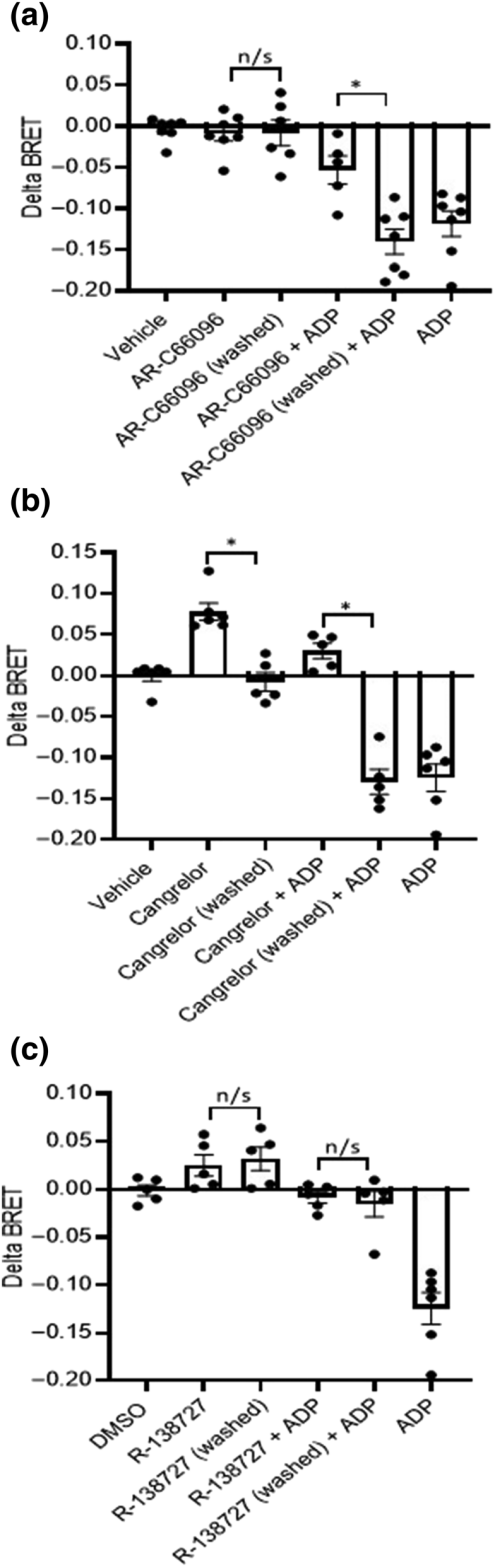
Prasugrel active metabolite but not cangrelor or AR‐C66096 activity at the P2Y_12_R is resistant to washout. HEK 293T cells were co‐transfected with human FLAG‐P2Y_12_R and heterotrimeric G proteins, RlucII‐Gαi, untagged Gβ and GFP‐Gγ. Forty‐eight hours after transfection, cells were treated with P2Y_12_R antagonist (AR‐C66096 [10 μM; a]), cangrelor (10 μM; b) or the active metabolite of prasugrel (1‐μM R‐138727; c) for 30 min at 37°C. Following antagonist treatment, cells were washed for 3 × 10 min, and receptor activity was compared with that following acute antagonist treatment alone (5 min). ADP‐stimulated (10 μM; 5 min) activity was assessed in non‐ticagrelor‐treated cells (ADP), in cells treated acutely with ticagrelor (Tic + ADP) or in cells following more prolonged ticagrelor treatment and subsequent washing (Tic washed + ADP). In (b), 2MeSADP‐stimulated (10 μM; 5 min) activity was assessed in non‐ticagrelor‐treated cells (2MeSADP), in cells treated acutely with ticagrelor (Tic + 2MeSADP) or in cells following more prolonged ticagrelor treatment and subsequent washing (Tic washed + 2MeSADP). Data shown are the means ± SEM of at least five independent experiments, each performed in triplicate. Statistical analysis was performed using one‐way ANOVA and followed by Bonferroni's multiple comparison test ([a] *P < 0.05 AR‐C66096 + ADP vs. AR‐C66096 washed + ADP; [c] *P < 0.05 cangrelor vs. cangrelor washed and *P > 0.05 cangrelor + ADP vs. cangrelor washed + ADP).

**FIGURE 4:**
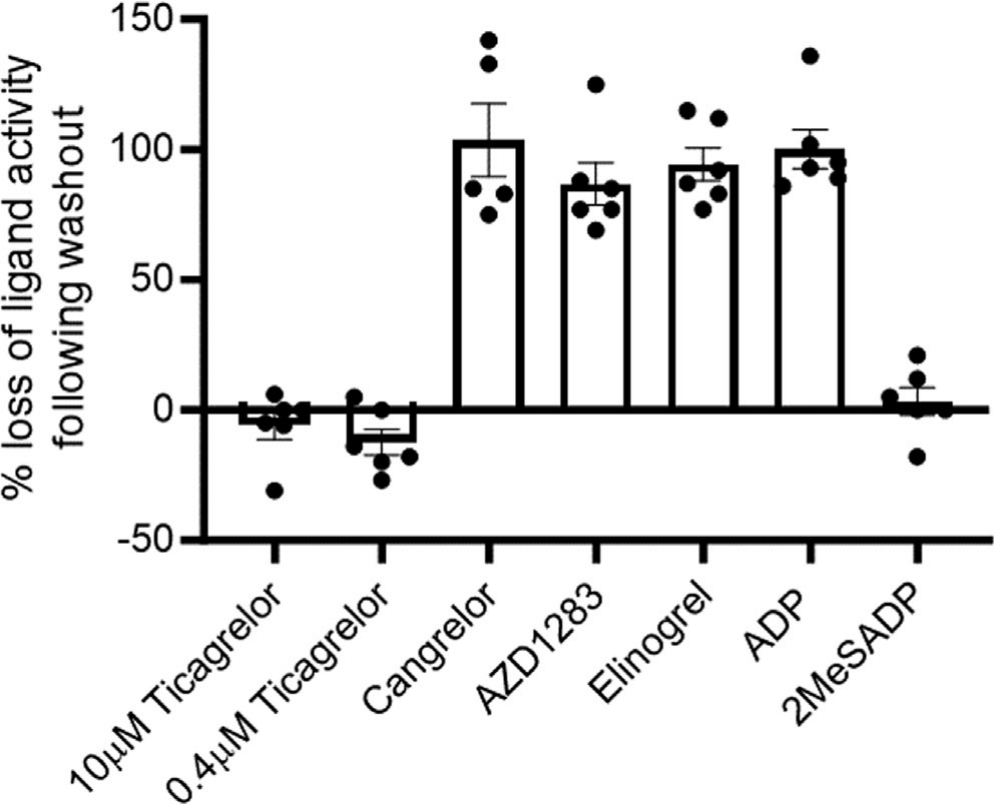
Ticagrelor and 2MeSADP activity at the P2Y_12_R is resistant to washout. HEK 293T cells were co‐transfected with human FLAG‐P2Y_12_R and heterotrimeric G proteins, RlucII‐Gαi, untagged Gβ and GFP‐Gγ. Forty‐eight hours after transfection, receptor activity (inverse agonist: ticagrelor, 0.4/10 μM; AZD1283, 10 μM; cangrelor, 10 μM; and elinogrel, 10 μM; agonist: ADP, 10 μM; 2MeSADP, 10 μM) was compared in cells treated with P2Y_12_R ligand (30 min) versus that in cells treated with ligand for 30 min followed by washout for 3 × 10 min. Data are expressed as % loss of P2Y_12_R ligand‐induced activity following washing and represent means ± SEM of at least five independent experiments, each performed in triplicate.

We next tested the reversibility of a range of P2Y_12_R antagonists and inverse agonists to ensure that our wash protocol was effective (Figure 3). As expected, pre‐treatment with the reversible P2Y_12_R antagonist AR‐C66096 (10 μM; 10 min) was able to effectively attenuate ADP‐stimulated P2Y_12_R activity (Figure 3a), an effect that was reversed by washing cells. Inverse agonism and antagonism of ADP‐stimulated P2Y_12_R activity by cangrelor pre‐treatment (10 μM; 10 min) also was reversed effectively by washing cells (Figure 3b). Pre‐treatment with the active metabolite of prasugrel (R‐138727; 10 μM; 10 min), an established irreversible P2Y_12_R antagonist, effectively blocked ADP‐stimulated P2Y_12_R activity, an effect that was not reversed by washing cells (Figure 3c). Further study focussing on the reversibility of the inverse agonist or agonist activity of the range of P2Y_12_R agonists and inverse agonists identified in Figure 1 revealed that the activity of ADP, AZD1283, cangrelor and elinogrel (all at 10 μM; 10 min) was lost following cell washing. Intriguingly, the activity of ticagrelor (10 and 0.4 μM) and the agonist 2MeSADP (10 μM) was resistant to cell washing (Figure 4).

**FIGURE 5:**
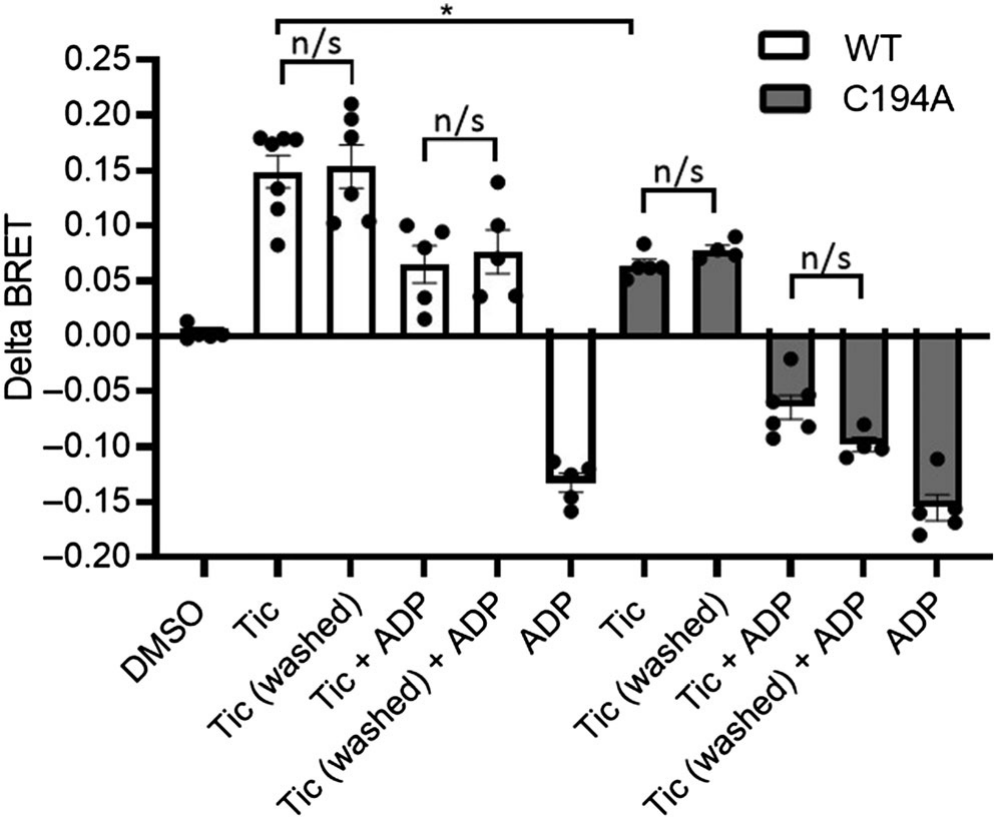
Resistance to washout of ticagrelor‐dependent activity is maintained in a P2Y_12_R mutant (C194A) displaying reduced ticagrelor activity. HEK 293T cells were transfected with either the human FLAG‐P2Y_12_R or FLAG‐C194A‐P2Y_12_R and heterotrimeric G proteins, RlucII‐Gαi, untagged Gβ and GFP‐Gγ. Forty‐eight hours after transfection, cells were treated with ticagrelor (10 μM) or vehicle control for 30 min at 37°C. Following ticagrelor treatment, cells were washed (Tic washed) for 3 × 10 min, and receptor activity was compared with that following acute ticagrelor treatment (10 μM; 5 min; Tic). ADP‐stimulated (10 μM; 5 min) activity was assessed in non‐ticagrelor‐treated cells (ADP), in cells treated acutely with ticagrelor (Tic + ADP) or in cells following more prolonged ticagrelor treatment and subsequent washing (Tic washed + ADP). Data shown are the means ± SEM of at least five independent experiments, each performed in triplicate. Statistical analysis was performed using one‐way ANOVA and followed by Bonferroni's multiple comparison test (*P < 0.05 ticagrelor response in FLAG‐P2Y_12_R vs. FLAG‐C194A‐P2Y_12_R).

One amino acid residue in P2Y_12_R identified as critical for ticagrelor activity is cysteine 194 (Hoffmann et al., 2014). We therefore investigated if this residue, in part, was responsible for the irreversibility of ticagrelor. As expected, inverse agonism of ticagrelor at the P2Y_12_R was significantly attenuated in a P2Y_12_R in which we mutated cysteine 194 to alanine (C194A; Figure 5). In addition, ticagrelor's ability to effectively antagonize ADP‐stimulated P2Y_12_R activity was significantly compromised in the C194A P2Y_12_R. Notably, the residual inverse agonism still present in C194A P2Y_12_R or antagonism of ADP‐stimulated P2Y_12_R activity still appeared resistant to washout.

**FIGURE 6:**
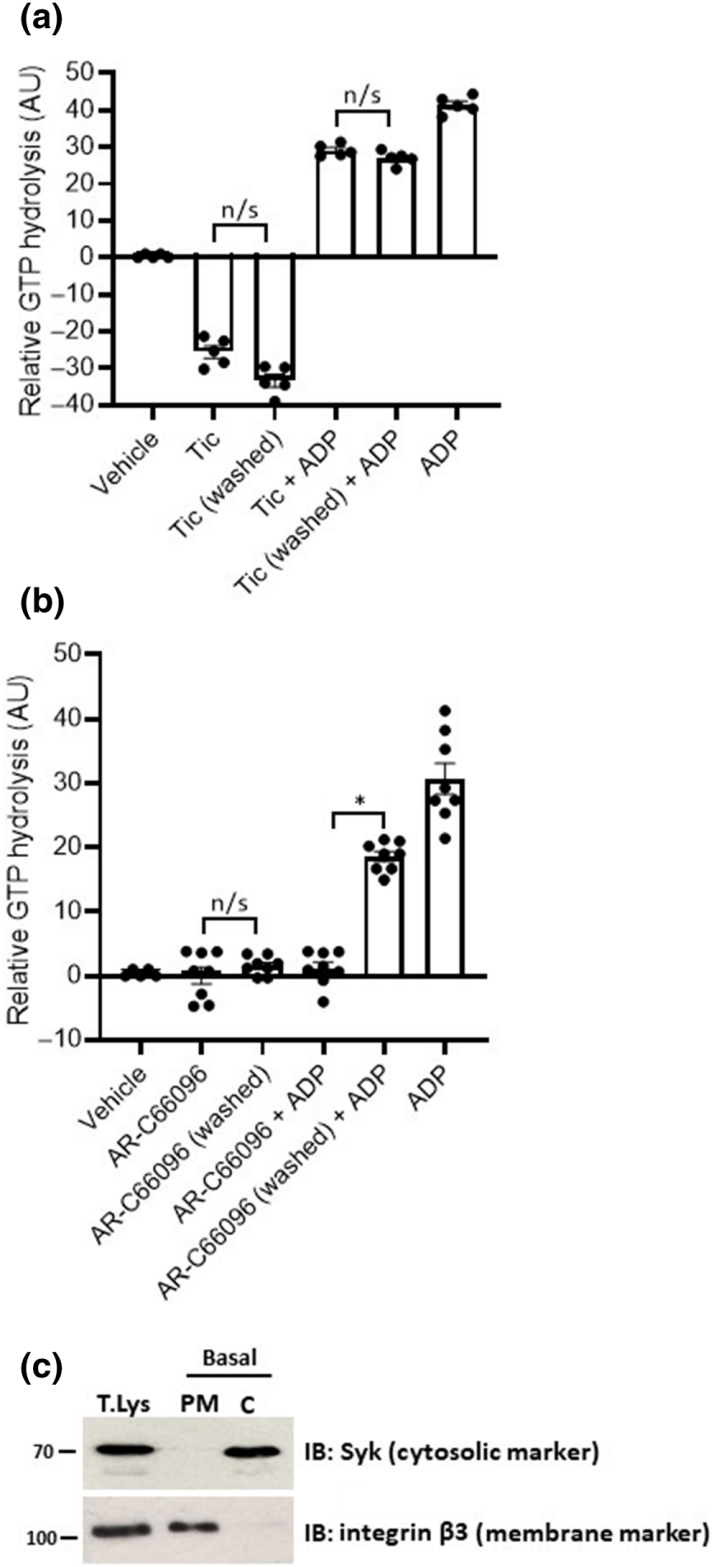
Ticagrelor‐dependent effects on P2Y_12_R activity are unaffected by washout in human platelets. Human washed platelets (1 × 10^9^/ml) were untreated or treated with (a) ticagrelor (10 μM) and (b) AR‐C66096 (10 μM) or vehicle (0.1% DMSO) for 30 min at 37°C. Platelets were then washed three times with intervals of 10 min before snap frozen in equal volume of fractionation buffer in liquid nitrogen. Membrane fractions were collected by ultracentrifugation. (c) The purity of our cell membrane preparation was assessed by western blot comparing the expression of platelet membrane (integrin β3) and cytosolic (Syk) expressed proteins in membrane versus cytosolic cell fractions. Platelet cell membranes were treated either with ADP (10 μM) or vehicle (0.1% DMSO) before incubation with recombinant GTP (2 μM) and DTT (1 mM) for 1 h at room temperature. GTPase‐Glo reagents were added to measure GTP hydrolysis. Data shown are the means ± SEM of at least five independent experiments. Statistical analysis was performed using one‐way ANOVA and followed by Bonferroni's multiple comparison test ([b] *P < 0.05 AR‐C66096 + ADP vs. AR‐C66096 washed + ADP).

Following on from our cell line studies, we next focussed on endogenous P2Y_12_R activity in human blood platelets. Our initial studies used a GTPase‐Glo assay to measure G‐protein activity following P2Y_12_R activation. As expected, stimulation of platelet cell membranes with ADP (10 μM) produced a pronounced increase in GTP hydrolysis indicative of increased P2Y receptor stimulation (Figure 6a,b). The purity of our cell membrane preparation was confirmed by western blot (Figure 6c) comparing the expression of platelet membrane (integrin β3) and cytosolic (Syk) expressed proteins in membrane versus cytosolic cell fractions (Joshi et al., 2018). The Immuno‐related procedures used comply with the recommendations made by the *British Journal of Pharmacology*. Pre‐treatment of platelets with either ticagrelor (10 μM; 30 min; Figure 6a) or AR‐C66096 (10 μM; 30 min; Figure 6b) effectively antagonized ADP‐stimulated rises in GTP hydrolysis. As in our cell line studies, and previously reported in human platelets (Aungraheeta et al., 2016), ticagrelor but not AR‐C66096 pre‐treatment effectively attenuated basal levels of platelet GTP hydrolysis indicative of reduced G‐protein activity and inverse agonist activity of ticagrelor at the P2Y_12_R. Neither antagonism of ADP‐stimulated or basal GTP hydrolysis by ticagrelor pre‐treatment was reversed by platelet washing (3 × 10‐min washes) prior to platelet membrane preparation (Figure 6a). AR‐C66096‐dependent antagonism of ADP‐stimulated GTP hydrolysis was readily reversed by platelet washing (Figure 6b).

**FIGURE 7:**
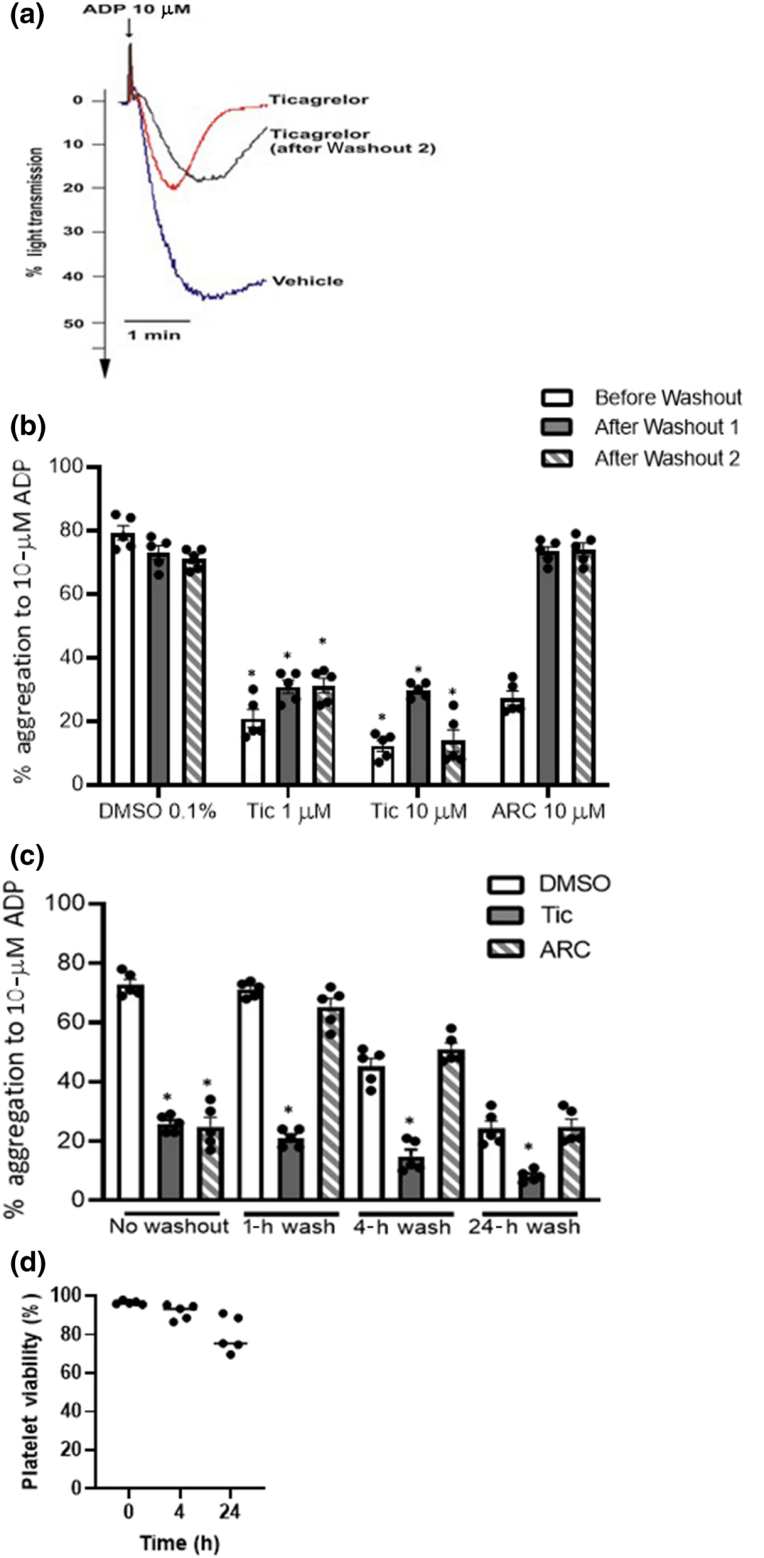
Inhibition of ADP‐stimulated platelet aggregation by ticagrelor is not reversed by extensive washout. (a, b) PRPs were treated either with vehicle (0.1% DMSO), ticagrelor (1 or 10 μM) or AR‐C66096 (10 μM) for 30 min at 37°C. Treated samples were either unwashed (before washout) or underwent either one 10‐min wash termed Washout 1 or two 10‐min washes termed Washout 2. As outlined in the methods following centrifugation steps, platelets were resuspended in PPP. In all cases, aggregation responses were recorded following the addition of ADP (10 μM) or vehicle (0.1% DMSO). (a) Representative aggregatory traces. (b) Data shown are the means ± SEM of at least five independent experiments. Statistical analysis was performed using one‐way ANOVA and followed by Bonferroni's multiple comparison test (*P < 0.05 DMSO vs. Tic [1 μM] or DMSO vs. Tic [1 μM] comparing before washout, after Washout 1 or after Washout 2). (c) As above, PRP was treated with vehicle (0.1% DMSO), AR‐C66096 (10 μM) or ticagrelor (10 μM) for 30 min at 37°C. Samples underwent one 10‐min wash and resuspended in PPP for 1, 4 or 24 h. Aggregation responses were subsequently recorded following the addition of ADP (10 μM) or vehicle (0.1% DMSO). (d) Platelet viability was assessed by measuring annexin V levels by FACs in platelet samples washed for 4 and 24 h as described in (c). Data shown are the means ± SEM of at least five independent experiments.

We next examined the reversibility of inhibition of ADP‐stimulated platelet aggregation by ticagrelor and AR‐C66096 (Figure 7). As shown in Figure 5a, pre‐treatment of human PRP with ticagrelor (1 μM; 30 min) effectively antagonized ADP (10 μM)‐stimulated platelet aggregation. This effect of ticagrelor was not reversed by extensive platelet washing (either one 10‐min wash termed Washout 1, or two 10‐min washes termed Washout 2) prior to ADP‐stimulated platelet aggregation (Figure 7a,b). AR‐C66096 block of ADP‐stimulated platelet aggregation was effectively reversed by washing (Figure 7b). To further probe our inability to washout ticagrelor, we further extended our second of the two washouts from 10 min to 1, 4 and 24 h (Figure 7c). ADP‐stimulated platelet aggregation began to significantly reduce following longer periods of wash (4 and 24 h) likely due to loss of P2Y receptor activity (Hardy et al., 2005), although platelet viability, as assessed by measuring annexin V levels, continued to remain >79% at the 24‐h washout time point (Figure 7d). Notably, however, ticagrelor antagonism of ADP‐stimulated platelet aggregation remained stubbornly resistant to reversal even following 24 h of wash, whereas that of AR‐C66096 was rapidly reversed within an hour. These studies confirmed our cell line studies, which indicated that ticagrelor appeared to show an irreversible mode of action at the P2Y_12_R versus the endogenous receptor agonist ADP.

**FIGURE 8:**
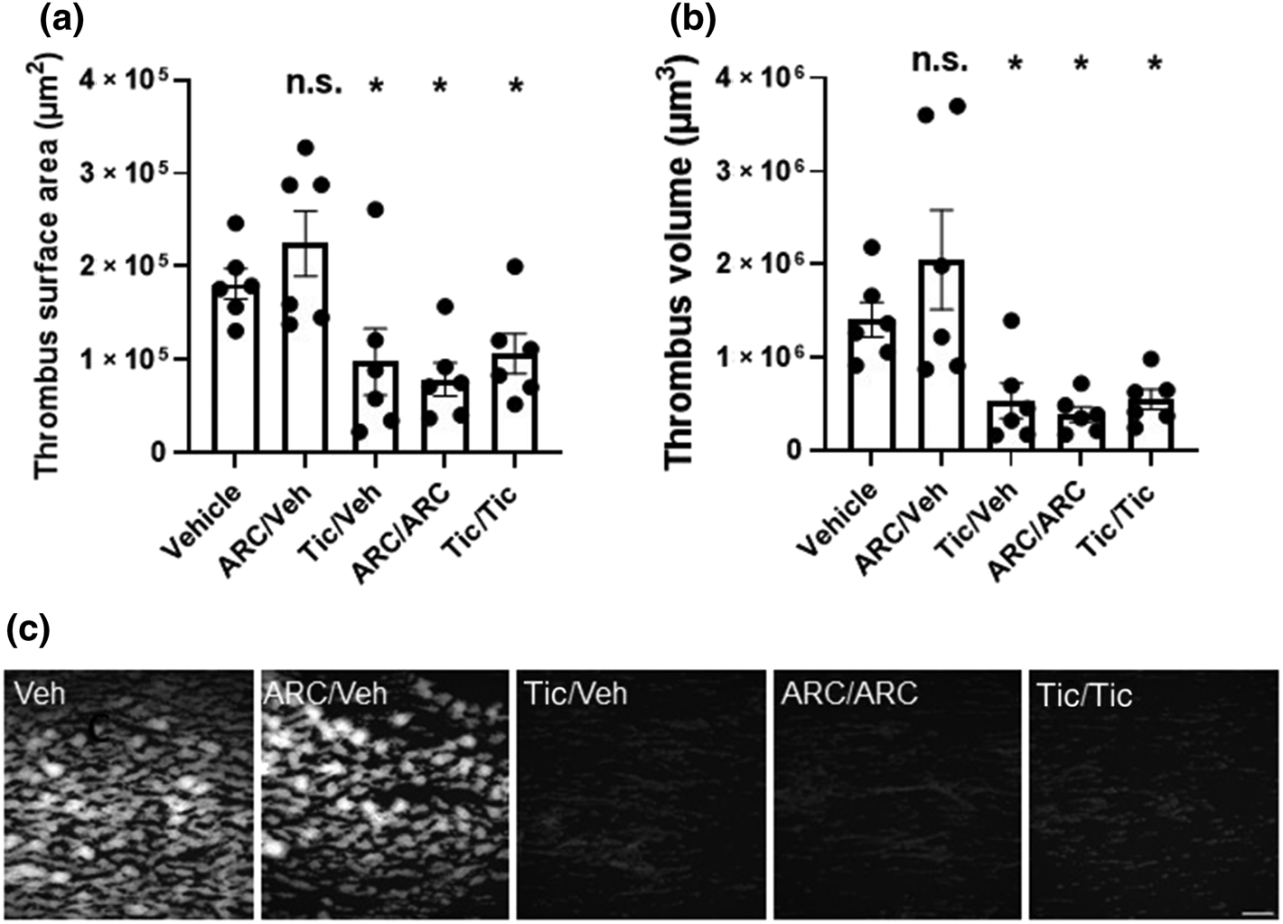
Ticagrelor is resistant to washout in platelets treated in whole blood. DMSO vehicle, AR‐C66096 (10 μM) or ticagrelor (10 μM) was added to whole blood for 10 min before centrifugation, washing and reconstitution of treated platelets into fresh, untreated plasma/red blood cells and re‐treated with vehicle, AR‐C66096 or ticagrelor as indicated for a further 10 min before flowing over collagen at arterial shear, fixation and visualization in order to monitor platelet deposition. (a) In vitro thrombus maximum projected surface area (n.s., not significant Veh vs. ARC/Veh; *P < 0.05 Veh vs. Tic/Veh; *P < 0.05 Veh vs. ARC/ARC; *P < 0.05 Veh vs. Tic/Tic). (b) In vitro thrombus volume (n.s., not significant Veh vs. ARC/Veh; *P < 0.05 Veh vs. Tic/Veh; *P < 0.05 Veh vs. ARC/ARC; *P < 0.05 Veh vs. Tic/Tic). (c) Representative images from a single *n*. Bars represent mean ± SEM from six independent experiments. Data were analysed by matched ANOVA with Dunnett's test comparing each treatment condition versus vehicle.

We further examined the reversibility of inhibition of platelet reactivity by ticagrelor and AR‐C66096 using an in vitro flow analysis model. In this model, platelets were flowed over a collagen‐coated slide at arterial shear with subsequent platelet deposition, an indicator of platelet reactivity. DMSO vehicle, AR‐C66096 or ticagrelor was added to whole blood for 10 min before fractionation, washing and reconstitution of treated platelets into fresh, untreated plasma/RBCs. These were then re‐treated either with vehicle, AR‐C66096 or ticagrelor as indicated for a further 10 min. As shown in Figure 8a, as expected, there was significant platelet deposition in DMSO‐treated controls. There was minimal deposition in both AR‐C66096 re‐treatment (ARC/ARC) and ticagrelor re‐treatment (Tic/Tic) samples indicative of effect inhibition of platelet P2Y_12_R activity, as reported by others when using this model. Platelet deposition was restored after washout in the ARC/Veh sample indicative of effective drug washout. In contrast, there was minimal platelet deposition after washout in the Tic/Veh sample. Analysis of the confocal images showed that there was a significant reduction in both thrombus surface area (*P* < 0.05) and thrombus volume (*P* < 0.05) in ticagrelor treatment after washout (Tic/Veh) compared to the vehicle (Figure 8b). However, there was no significant difference in thrombus surface area (*P* = 0.519) and thrombus volume (*P* = 0.454) in AR‐C66096 treatment after washout (ARC/Veh) compared to the vehicle (Figure 8b). There were significant reductions in both thrombus surface area and thrombus volume in AR‐C66096 re‐treatment (ARC/ARC) and ticagrelor re‐treatment (Tic/Tic). These data further reiterated in a more physiologically based assay that ticagrelor appeared to show an irreversible mode of action at the P2Y_12_R.

**FIGURE 9:**
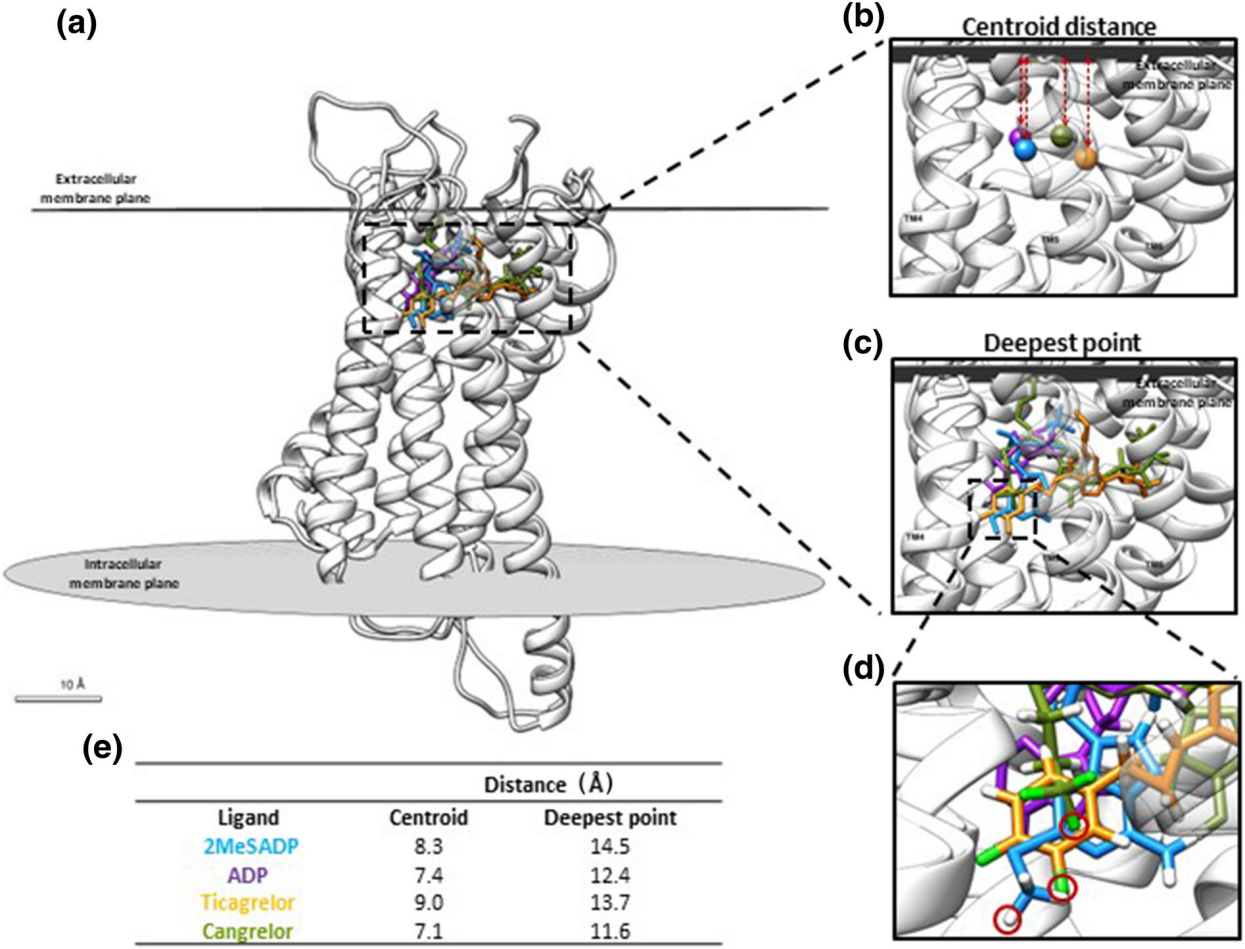
In silico docking reveals that ticagrelor and 2MeSADP penetrate more deeply into the orthosteric binding pocket of the P2Y_12_R than cangrelor. (a) Overlay of the agonist and antagonist models of the P2Y_12_R with 2MeSADP (blue), ADP (purple), ticagrelor (orange) and cangrelor (green) docked within the receptor orthosteric site. Model also shows the outmost plane of both the extracellular and intracellular membranes. The 2MeSADP binding pose is based on reported crystal structure, whilst that for ADP, ticagrelor and cangrelor is based on simulated docking results. (b) Geometric centre (centroid) of each ligand shown. Centroid distance to the extracellular membrane for each ligand calculated and summarized in (e). (c) Deepest point for each ligand identified and distance to extracellular membrane plane calculated and displayed in (e). (d) Focus zoom in of residues of 2MeSADP, ticagrelor and cangrelor identified to show deepest penetration into orthosteric binding pocket. (e) Summary of distances for the deepest penetration points and ligand centroid from the extracellular membrane plane for each ligand.

To further understand why both ticagrelor and 2MeSADP appeared resistant to washout, we performed in silico docking and all‐atom MD simulations of P2Y_12_R and compared the binding profile of the agonists ADP and 2MeSADP and inverse agonists ticagrelor and cangrelor. Employing both the agonist‐ and antagonist‐bound crystal structures of P2Y_12_R, agonists were docked within the agonist model, antagonists in the antagonist model (Figure 9). Based on the suggested binding poses, ticagrelor, 2MeSADP and cangrelor occupy a binding pocket within the orthosteric site, which is distinct to that of ADP (Figure 9a). This is in accordance with previously published data (Zhang, Zhang, Gao, Paoletta, et al., 2014; Zhang, Zhang, Gao, Zhang, et al., 2014). The geometric centre (centroid) of each ligand was estimated, and interestingly, cangrelor was found to sit higher in the receptor orthosteric cavity when compared to the other ligands (Figure 9b). The deepest penetration point for each ligand was identified, and distance to extracellular membrane plane was calculated (Figure 9c–e). Intriguingly, we predict that the wash‐resistant compounds ticagrelor and 2MeSADP penetrate more deeply into the binding pocket than cangrelor.

## 4 DISCUSSION

The mechanism of action of ticagrelor is distinct from that of previously described antiplatelet agents targeting P2Y_12_R (Aungraheeta et al., 2016; Hoffmann et al., 2014; Van Giezen et al., 2009). Ticagrelor has been demonstrated to be an inverse agonist at the platelet P2Y_12_R (Aungraheeta et al., 2016; Garcia et al., 2019). In addition, ticagrelor has been shown to inhibit the platelet adenosine ENT1 transporter (SLC29A1), resulting in the accumulation of extracellular adenosine that further dampens down platelet reactivity (Aungraheeta et al., 2016). The central aim of this current study was to further elucidate the mode of action of ticagrelor antiplatelet therapy focussing predominantly on drug reversibility, which is crucially important to consider when assessing the safety and efficacy of pharmacological therapeutics.

Given recent clinical studies (Godier et al., 2015; Trenk et al., 2019; Willeman et al., 2018; Zhang et al., 2019), we sought to probe the reversibility of ticagrelor binding to P2Y_12_R in comparison with other receptor antagonists. Ticagrelor has demonstrated clinical superiority over many antiplatelet agents (Bonaca et al., 2015; Wallentin et al., 2009). However, the significant side effect of spontaneous major bleeding, and bleeding during invasive procedures as with other P2Y_12_R antagonists, remains with ticagrelor. In respect to the thienopyridines, clopidogrel and prasugrel, the cause for this unwanted bleeding is thought in part to be due to their mode of action and irreversible blockade of the P2Y_12_R. These drugs effectively attenuate platelet function for the duration of their lifespan. There is a clear need therefore for reversible P2Y_12_R antagonists especially in the context of patients undergoing emergency invasive procedures or requiring abrupt cessation secondary to significant bleeding. Our study demonstrates that the endogenous agonist ADP was unable to restore P2Y_12_R activity following ticagrelor treatment even after extensive washing either in cell lines or human platelets.

Although ticagrelor has been licensed as the first perorally active and reversible P2Y_12_R antagonist, evidence contests ticagrelor's reversibility at P2Y_12_R (Gerrits et al., 2017). For example, ticagrelor displayed a similar bleeding profile to clopidogrel during invasive procedures (James et al., 2009), where ticagrelor was withheld for 24–72 h and clopidogrel for 5 days. More recent studies indicate that platelet supplementation via transfusion does not rescue platelet inhibition resulting from ticagrelor action (Godier et al., 2015; Trenk et al., 2019; Willeman et al., 2018; Zhang et al., 2019). Potentially, this is because ticagrelor and its active metabolite (AR‐C124910XX) have much longer half‐lives (9 and 12 h, respectively) than the thienopyridine derivatives prasugrel and ticagrelor, with their ongoing presence able to inhibit fresh platelets at the time of the transfusion (Butler & Teng, 2010; Cave et al., 2019; Zhu et al., 2019). Intriguingly, ticagrelor‐targeted monoclonal antibody fragments have been engineered, appearing efficacious in the rapid reversal of the drug’s actions (Bhatt et al., 2019). The data from these studies ultimately underscore the inconsistencies in the reported reversibility of ticagrelor.

Our study is the first, to our knowledge, to demonstrate that ticagrelor's inverse agonism and antagonism of ADP‐stimulated P2Y_12_R activity are not readily reversed in cell lines or more importantly human blood platelets. Previous seminal studies by Van Giezen et al. (2009) have demonstrated that [^3^H]‐ticagrelor binds to the P2Y_12_R in a reversible manner with a *t*_1/2_ (on) and *t*_1/2_ (off) of 3.8 ± 0.9 and 13.5 ± 1.9 min, respectively. These studies were undertaken in P2Y_12_R‐transfected Chinese hamster ovary (CHO)‐K1 membranes, and as is standard for such studies, [^3^H]‐ticagrelor (40 nM) was displaced with unlabelled ligand (10 μM). Clearly, therefore, ticagrelor is not a classical irreversible antagonist at P2Y_12_R like the thienopyridines, clopidogrel and prasugrel. Intriguingly, our studies have demonstrated that ticagrelor activity was maintained following extensive washing, in our whole‐cell studies (BRET and platelet aggregation/adhesion) as well as cell membrane studies (GTPase‐Glo assays). Preliminary studies in CHO cells expressing P2Y_12_R have shown that ticagrelor shows little demonstrable reversibility versus ADP.

In agreement with Van Giezen et al. (2009) and consistent with our previous studies (Aungraheeta et al., 2016), we showed that 2MeSADP, a P2Y_12_R agonist, with a 100‐fold higher potency than ADP, was more readily able to compete with ticagrelor. Again, as with ADP, wash steps did not reverse residual ticagelor antagonism of 2MeSADP. We (Aungraheeta et al., 2016) and others (Van Giezen et al., 2009) have demonstrated that ticagrelor shows non‐competitive antagonism versus ADP at therapeutic antagonist concentrations. Ticagrelor is suggested to bind to a site to that distinct from ADP on P2Y_12_R and act through a non‐competitive, allosteric mechanism to prevent ADP‐stimulated receptor activation. Previous mutagenesis analysis has shown that cysteine 194 of the P2Y_12_R plays a key role in coordinating ticagrelor binding (Hoffmann et al., 2014). Interestingly, we found that the resistance of ticagrelor to removal by washing was maintained in a P2Y_12_R mutant (C194A) with demonstrably reduced ticagrelor activity.

Notably, Gerrits et al. (2017) reported that prolonged incubation of ex vivo blood platelets with ticagrelor (24 h) resulted in incomplete reversibility of platelet reactivity, a phenomenon not observed after shorter periods of exposure of ticagrelor. This study suggested that the process of irreversible inhibition was time dependent. Our study in cell lines and human platelets indicates a more rapid emergence of irreversible inhibition. The possibility of ticagrelor acting at a different off‐target site to cause irreversible platelet inhibition cannot be excluded although would seem less plausible in our HEK 293T cell line system. Given the short time periods of ticagrelor treatment (30 min or less), we would suggest that changes in protein expression are unlikely to explain the apparent irreversibility of ticagrelor reactivity. Importantly, we show that ticagrelor's resistance to washing is retained at therapeutically relevant plasma concentrations of drug (0.4 μM). Potentially, ticagrelor binding may alter the P2Y_12_R conformation or more likely some ticagrelor may remain bound to P2Y_12_R. Intriguingly, the SWAP‐2 study demonstrated a failure of prasugrel to significantly block P2Y_12_R function when administered 36 and 60 h after the last ticagrelor dose (Angiolillo et al., 2014). This would support the theory that residual ticagrelor binding or ticagrelor‐dependent changes in P2Y_12_R conformation prevent interaction with prasugrel's active metabolite.

Our studies suggest that ticagrelor is not the only P2Y_12_R ligand to show significant resistance to reversal following washing. Notably, 2MeSADP, a potent agonist at the P2Y_12_R, appeared resistant to washout. Unfortunately, comparison of the structure of 2MeSADP with that of ticagrelor or the more reversible P2Y_12_R ligands fails to give any obvious structure/function or chemical differences (for example, ligand lipophilicity), to indicate why these two compounds appear resistant to washout.

Although the resolution of the P2Y_12_R by X‐ray crystallography provided significant insights into its structure (Zhang, Zhang, Gao, Paoletta, et al., 2014; Zhang, Zhang, Gao, Zhang, et al., 2014), including the presence of two binding pockets, there is still no definitive binding pose for ticagrelor at the P2Y_12_R. An extensive molecular docking study of the major classes of substances, previously reported as P2Y_12_R ligands, was unable to dock ticagrelor to the agonist‐bound P2Y_12_R structure. A ‘hybrid’ receptor for successful ticagrelor docking was required, which resembled the agonist‐bound P2Y_12_R except for the top portion of TM6, which was taken from the antagonist‐bound P2Y_12_R structure (Paoletta et al., 2015). We also performed in silico docking and all‐atom MD simulations of the P2Y_12_R and compared the binding profile of the agonists ADP and 2MeSADP and inverse agonists ticagrelor and cangrelor. Employing both the agonist‐ and antagonist‐bound crystal structures of the P2Y_12_R, agonists were docked within the agonist model and antagonists in the antagonist model. We found that such a protocol did not require the design of a ‘hybrid’ receptor. Interestingly, we found that cangrelor sits higher and penetrates less deeply into the receptor orthosteric cavity when compared to the wash‐resistant ticagrelor and 2MeSADP. Further extensive mutagenesis studies, beyond the scope of this work, may help define how the potential deeper penetration of these ligands into the binding pocket may relate to their wash resistance.

In conclusion, our study highlights incomplete reversibility of platelet and P2Y_12_R inhibition following exposure to ticagrelor. This has obvious and clear clinical implications for patients requiring surgical intervention following ticagrelor therapy and underscores current guidelines, which state that in patients on P2Y_12_R antagonists who need to undergo non‐emergency major non‐cardiac surgery, postponing surgery for at least 5 days after cessation of ticagrelor should be considered if clinically feasible and unless the patient is at high risk of ischaemic events.

## AUTHOR CONTRIBUTIONS

**Jawad Khalil**: Conceptualization (equal); data curation (equal); formal analysis (equal); investigation (lead); methodology (equal); writing—original draft (equal). **Tudor Dimofte**: Investigation (equal); methodology (equal). **Timothy Roberts**: Investigation (equal); methodology (equal). **Michael Keith**: Investigation (supporting); methodology (supporting). **Kumuthu Amaradasa**: Formal analysis (supporting); investigation (supporting). **Matthew S. Hindle**: Formal analysis (supporting); investigation (supporting). **Sukhinder Bancroft**: Investigation (equal); methodology (equal). **James L. Hutchinson**: Investigation (equal); methodology (equal). **Khalid Naseem**: Funding acquisition (supporting); writing—review and editing (equal). **Thomas Johnson**: Writing—review and editing (equal). **Stuart J. Mundell**: Conceptualization (equal); funding acquisition (lead); project administration (lead); writing—original draft (lead); writing—review and editing (lead).

## CONFLICT OF INTEREST STATEMENT

None.

## DECLARATION OF TRANSPARENCY AND SCIENTIFIC RIGOUR

This Declaration acknowledges that this paper adheres to the principles for transparent reporting and scientific rigour of preclinical research as stated in the *BJP* guidelines for Design & Analysis and Immunoblotting and Immunochemistry and as recommended by funding agencies, publishers and other organizations engaged with supporting research.

## Data Availability

The data that support the findings of this study are available from the corresponding author upon reasonable request. Some data may not be made available because of privacy or ethical restrictions.
